# Detection of *Aeromonas hydrophila* in Liquid Media by Volatile Production Similarity Patterns, Using a FF-2A Electronic Nose

**DOI:** 10.3390/s130100736

**Published:** 2013-01-07

**Authors:** Kouki Fujioka, Eiji Arakawa, Jun-ichi Kita, Yoshihiro Aoyama, Yoshinobu Manome, Keiichi Ikeda, Kenji Yamamoto

**Affiliations:** 1 Department of Molecular Cell Biology, Institute of DNA Medicine, Research Center for Medical Sciences, The Jikei University School of Medicine, Tokyo 105-8461, Japan; E-Mails: kfujioka@jikei.ac.jp (K.F.); manome@jikei.ac.jp (Y.M.); ikedak@jikei.ac.jp (K.I.); 2 Department of Bacteriology, National Institute of Infectious Diseases, Tokyo 162-8640, Japan; E-Mail: earakawa@nih.go.jp; 3 Research & Development Group, Business Development Department, Analytical & Measuring Instruments Division, Shimadzu Corporation Ltd., Kyoto 604-8442, Japan; E-Mails: j-kita@shimadzu.co.jp (J.K.); aoyama@shimadzu.co.jp (Y.A.); 4 Research Institute, National Center for Global Health and Medicine, Tokyo 162-8655, Japan

**Keywords:** *Aeromonas hydrophila*, pre-screening, rapid detection, volatile production patterns, electronic nose, FF-2A

## Abstract

A technique for rapid detection of pathogenic microorganisms is essential for the diagnosis of associated infections and for food safety analysis. *Aeromonas hydrophila* is one such food contaminant. Several methods for rapid detection of this pathogen have been developed; these include multiplex polymerase chain reaction assays and the colony overlay procedure for peptidases. However, these conventional methods can only be used to detect the microorganisms at high accuracy after symptomatic onset of the disease. Therefore, in the future, simple pre-screening methods may be useful for preventing food poisoning and disease. In this paper, we present a novel system for the rapid detection of the microorganism *A. hydrophila* in cultured media (in <2 h), with the use of an electronic nose (FF-2A). With this electronic nose, we detected the changes of volatile patterns produced by *A. hydrophila* after 30 min culture. Our calculations revealed that the increased volatiles were similar to the odours of organic acids and esters. In future, distinctive volatile production patterns of microorganisms identified with the electronic nose may have the potential in microorganism detection.

## 1. Introduction

Electronic noses have been used for various purposes [1], including chemical sensing [2–6], microorganism profiling [7–11], disease diagnosis [12–15], and quality analyses for foods and beverages [16–20]. In microorganism profiling studies, for example, we showed that the electronic nose (FF-2A, Shimadzu Corporation, Kyoto, Japan) could detect the volatile concentration changes depending on the number of yeast between 10^2^ and 10^7^ colony forming units (CFU)/mL in 90 min [11]. Another group demonstrated that the electronic nose system can recognize volatile production patterns of pathogens including *Klebsiella pneumoniae, Pseudomonas aeruginosa*, and *Escherichia coli* at the species level [10]. They detected the microorganisms' volatile patterns after 24 h incubation (preincubation concentrations: more than 1.0 × 10^5^ CFU/mL).

Among such bacterial families, *Aeromonas* species are facultative anaerobic Gram-negative bacteria that belong to the *Aeromonadaceae* family [21]. These bacteria are found in water, soil, and food items [22]. Some species can grow in chlorinated water [23]. The US Environmental Protection Agency has included *Aeromonas hydrophila* in the list of potential contaminant organisms that will require regulation in the future, as per the Safe Drinking Water Act [24]. In recent years, *Aeromonas* species have been isolated from patients with diarrhoea, especially children aged <5 years [25]. Although diarrhoea caused by *Aeromonas* species is often mild, it can occasionally become serious, accompanied by haematochezia, abdominal pain, and fever [26]. Moreover, although a link between diarrhoea and the consumption of food contaminated with *Aeromonas* has not been conclusively identified, putative virulence factors have been detected in contaminated food or clinical isolates [25,27,28]. Other diseases associated with these bacteria are wound infections, pneumonia, meningitis, and septicaemia in humans [29,30]. Some patients with *Aeromonas* infections have developed acute illness and died of septic shock [31]. *A. hydrophila* and *A. sobria* are often isolated from patients with diarrhoea and the abovementioned diseases [26].

In clinical practice, microorganisms in patient samples are mainly identified by isolation from a selective agar medium or by enzyme-substrate reactions. Although these methods enable definitive identification of contaminant microorganisms, they require long periods for the processing and detection. Recently, multiplex polymerase chain reaction (PCR) assays [32,33] and the colony overlay procedure for peptidases (COPP) have been proposed for the rapid detection of *Aeromonas* species [34]. PCR assays have been used to determine the distribution of virulence factors among *Aeromonas* species isolated from drinking water utilities [32] and from a trout farm [33]. It was found that bacterial isolation on agar plates required 24–60 h, while the PCR method was completed in less than 2 h. Further, COPP has been used to identify *A. hydrophila* on the basis of its lysyl aminopeptidase activity; in this assay, the culture plates were incubated for 16–18 h, after which enzyme activity was detected within 10 min [34]. Both the PCR assays and the COPP are rapid, sensitive, and accurate.

However, these techniques aim at detecting *Aeromonas* species at high accuracy after disease occurrence. For preventing food or water-borne illness, other quick tests may be useful for determining the presence of *Aeromonas* species. Thus we propose a novel rapid method based on the combination of an electronic nose and odour descriptors for simple identifying microorganisms, based on the analysis of the odours arising from volatile compounds released from microorganism metabolism. Especially, *A. hydrophila* is reported to metabolize many types of carbohydrates to organic acids [35,36]. We hypothesized these volatile compounds may be able to be detected with an electronic nose.

In this study, we investigated the possibility of applying the FF-2A electronic nose for the rapid detection of *A. hydrophila* in liquid culture media. This electronic nose is equipped with 10 metal oxide semiconductor sensors and a preconcentration tube, consisting of carbon-based adsorbents to eliminate water vapour, which affects the sensors. Therefore, this device yields measurements with high sensitivity and reproducibility [37,38]. In order to know the differences of the detected odours, we compared them with known odours of several standard gases.

## 2. Experimental Section

### 2.1. Microorganisms and Culture Conditions

*A. hydrophila* was isolated from a water sample obtained in Japan. The bacteria were cultured in lysogeny broth (LB; Merck, Darmstadt, Germany; containing 1% peptone derived from casein, 0.5% yeast extract, and 1% NaCl) or modified nutrient broth (Becton Dickinson, Franklin Lake, NJ, USA; containing Bacto beef extract and Bacto peptone) supplemented with 1% NaCl. The starter culture was inoculated at a concentration of 9.6 × 10^2^ CFU/mL and incubated at 37 °C for 0–120 min. Samples of the media containing *A. hydrophila* were collected at specific time points and filtered through a 0.45 μm filter to remove the bacteria.

### 2.2. Measurement and Calculation of Volatile Compounds and Standard Gases

Aliquots of the liquid samples (2 mL, containing *A. hydrophila* and the media) were collected into 2 L polyethylene terephthalate (PET) bags filled with dry nitrogen. The samples in the bags were allowed to equilibrate at 25 °C for 30 min. Further, 200 mL of headspace volatiles were collected, transferred to new 2 L PET bags, diluted with dry nitrogen, and equilibrated at 25 °C for 30 min. These diluted samples were introduced into a trap tube of the FF-2A electronic nose (Shimadzu Corporation, Kyoto, Japan) for 60 s and then exposed to a sensor array with pure nitrogen gas. All the samples were measured twice, and the mean data are presented. The first data was obtained within approximately 80 min after the culture started.

**Figure 1.**
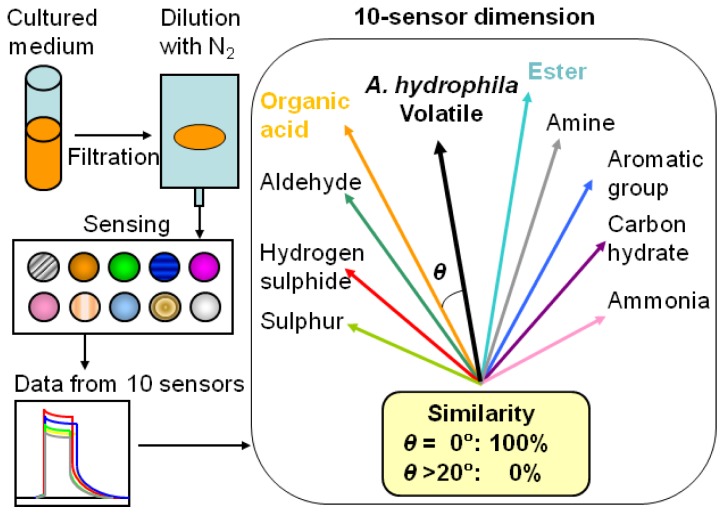
Scheme of measurement and calculation method. The similarities were calculated by the FF-2A algorithm (see the Experimental section) using the angles between the vectors.

Odour similarities were calculated with the FF-2A software in the following manner: the device contains 10 metal oxide semiconductor sensors that vary in their sensitivity and selectivity for different odorous substances [5,11,38]. These sensors were standardized with nine gases (hydrogen sulphide, methyl mercaptan, ammonia, trimethylamine, propionic acid, butyraldehyde, butyrl acetate, toluene, and heptane). These gases were decided as the standard materials from the environmental offensive odours which are targeted to cover general odour problems in Japan. On the basis of the signals yielded by the sensors, standard vectors corresponding to the nine standard gases were calculated in a 10-sensor dimension (Figure 1). The produced volatile samples from the culture media were measured and compared with the standard gases' vectors. Finally, the data were presented in terms of the similarities between the two data sets, calculated using the angles between the sample vectors. This calculation system based on angles is unique and useful to compare the volatile specificities easily, even between different concentrations [37]. Therefore, it is more suitable for gaining specificities from unknown-concentration samples than multivariate statistics applied only on the raw data. For this calculation, the following criteria were used: θ = 0°, 100% similarity; θ > 20°, 0% similarity. These algorithms were based on the following data from developers' measurement results: at θ = 0°, humans perceive the odour similarly (100% similarity), while at θ > 20°, humans perceive the odour differently [37]. The approximated curves and correlation factors (similarity *vs.* culture time) in the figures were calculated using Microsoft Excel 2003.

## 3. Results and Discussion

**Figure 2.**
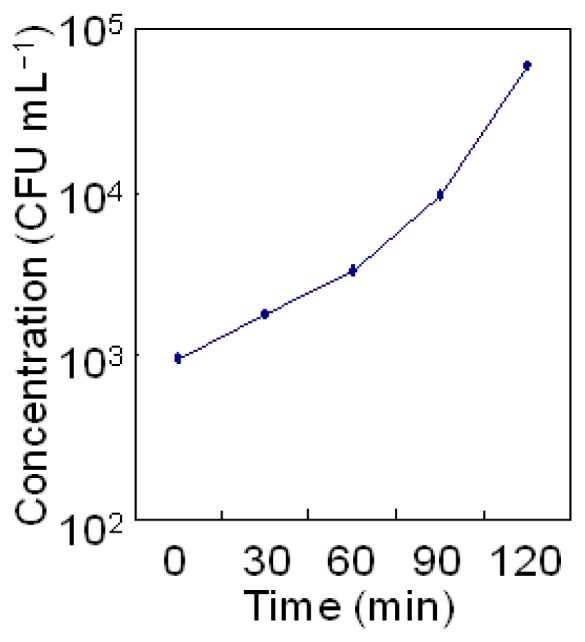
Growth curve of *A. hydrophila* from 0 to 120 min of culture.

The growth curve of *A. hydrophila* against time is shown in Figure 2. The starter culture containing 9.6 × 10^2^ CFU/mL of the bacteria was incubated at 37 °C in LB; the bacterial count increased to 1.8 × 10^3^ CFU/mL after 30 minutes, 3.3 × 10^3^ CFU/mL after 60 min, 9.6 × 10^3^ CFU/mL after 90 min, and 6.0 × 10^4^ CFU/mL after 120 min.

**Figure 3.**
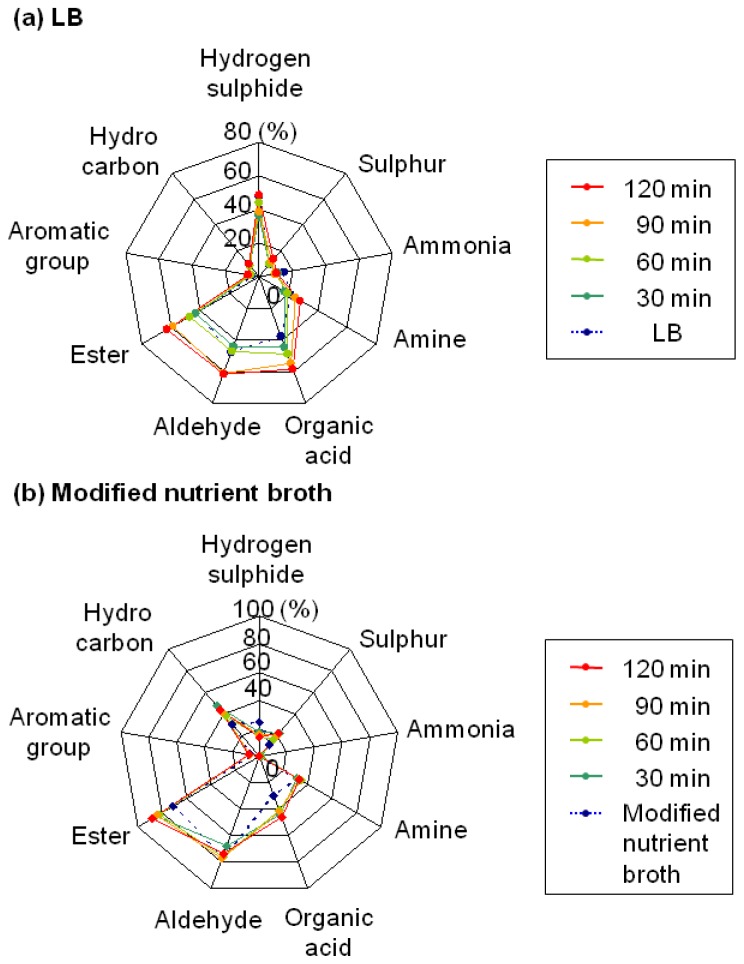
Similarity charts showing the volatile production patterns of *A. hydrophila* against 9 standard gases over the indicated culture time. Volatile production pattern of *A. hydrophila* in LB (**a**) or a modified nutrient broth (**b**). In the FF-2A algorithm, 100% similarity indicates an odour that humans perceive in the same manner, and 0% similarity indicates one that humans perceive differently (see the Experimental section). The measurements were conducted twice, and the mean data are shown.

Figure 3 shows the similarities in the odours arising from the *A. hydrophila* derived volatiles against those arising from the nine standard gases. The first data were collected in 80 min from the cultured sample collection. In order to determine the differences in the volatiles released on culture in different media, we used two liquid media—LB (containing yeast extract) and a modified nutrient broth (containing beef extract).

For LB cultures, the similarities between the volatiles from *A. hydrophila* and organic acid markedly increased with time from 38.2 to 58.4% (Figure 3(a)). Further, the similarities with the ester group also increased drastically from 43.4 to 62.9%. On culture in the modified nutrient broth, the similarities of the volatiles with the organic acids (30.0 to 46.1%) and esters (70.8 to 87.9%) also increased. Both the similarity charts indicated that minor modifications in the culture medium did not affect the tendency for increased production of distinctive odours.

Previous studies have revealed that *A. hydrophila* strains produce several acids from various types of carbohydrates [35,36]. Abbot *et al.* investigated fermentation reactions of 17 kinds of carbohydrates and detected acids from 11. Furthermore, Lee *et al.* showed the time-dependent changes in the concentrations of volatile fatty acids (acetate and formate) from carbohydrate fermentation.

The increase of the similarities observed for organic acids and esters was almost compatible with these previous studies in chemical categories and may become distinguishing markers of the presence of *A. hydrophila.* Moreover, volatile odours may be described objectively as the combination of distinctive odour descriptors from organic acids and ester. The former was described as pungent, sour, and sweaty [39], the latter was described as green, pungent, and sweet [40].

**Figure 4.**
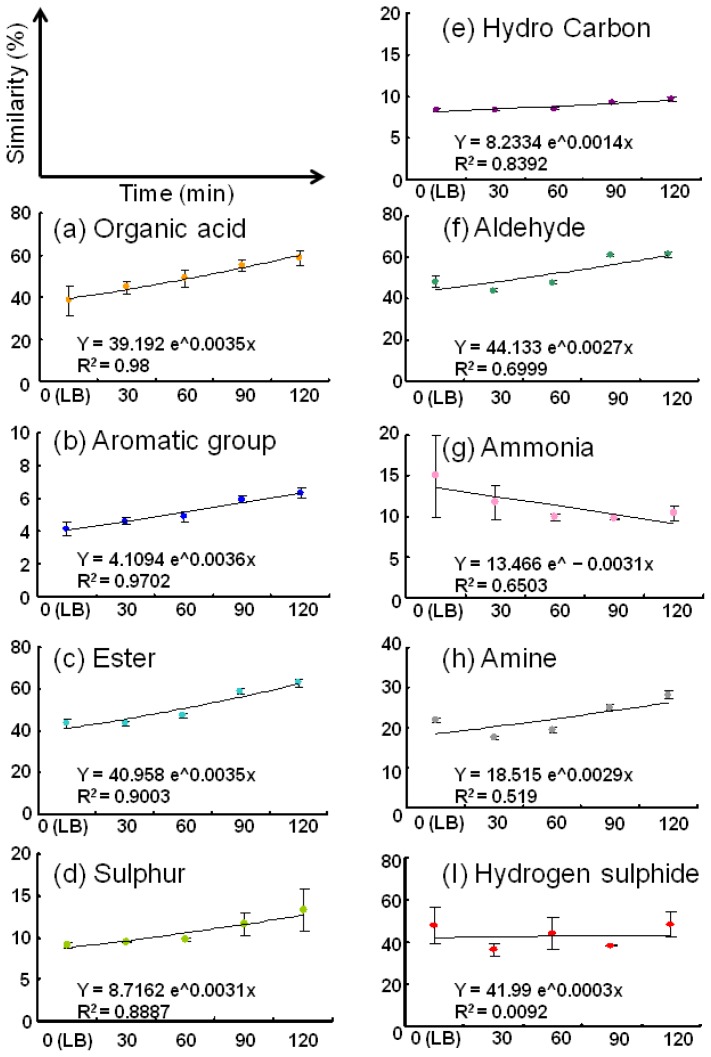
Time-dependent changes in the similarities between the volatiles and each type of standard gas. The volatile production patterns of *A. hydrophila* in LB are shown. The approximated curves and correlation factors were calculated using Microsoft Excel 2003. The graphs were categorized on the basis of the correlation factors. The measurements were conducted twice, and the mean ± standard deviation data are shown.

In order to identify the differences in greater detail, we plotted a similarity graph of the odours of the volatiles released on culture in LB against the odour of each standard gas (Figure 4). The graphs were categorized in terms of the correlation factors. As mentioned earlier, the similarities of the volatiles with organic acids (a) and esters (c) increased markedly with time. In addition, the correlation factors for both these standards were high (0.98 and 0.90, respectively). On the other hand, the similarities of the volatile compounds with the aromatic group (b), sulphur (d), and hydrocarbon (e) did not increase considerably, but the correlation factors were high. Other groups (f–h) showed low correlation factors, probably because these standard gases may not be suitable for detecting the metabolic products in this case.

Presence of these distinctive odour groups suggested the possibility of presence of *A. hydrophila*. Further, since the detected odours were correlated with the concentration of *A. hydrophila*, this method can be useful for determining the concentration of bacteria in unknown samples. The modified nutrient broth, on the other hand, had relatively low correlation factors (0.0261–0.76, data not shown). These results suggest that LB is more suitable for the detection of *A. hydrophila* with the electronic nose on the basis of volatiles released. The differences of volatiles between the two media may affect the detection ability of the electronic nose.

The sensitivity, efficiency, and specificity of the electronic nose render it highly advantageous for the detection of microorganisms. First, the sensitivity of the electronic nose used in the present study can be attributed to the fact that it was equipped with the preconcentration tube for eliminating water vapour, which affects the activity of the semiconductor sensors [37]. Preconcentration tubes also enable reproduction of the results under varied humidity conditions.

Second, the electronic nose used was efficient since it took less than 2 h to obtain the first data (incubation time, 30 min; measurement time, 80 min). Moreover, the measurement time included dilution of sample volatiles by two natural equilibration steps to dilute the volatiles to appropriate concentrations (See Section 2) The natural equilibration of the volatiles accounted for 30 min (60 min in total); thus, the process time could be reduced with the development of a more rapid equilibration method.

Finally, to determine the specificity of the system, more volatile data from other microorganisms are needed. Although *A. hydrophila* was the sole pathogen demonstrated in this study, data on the volatile compounds produced by other microorganisms will enable rapid detection and classification of these microorganisms. Moreover, selection of appropriate standard gases and sensors will be able to carry out more rapid screening.

## 4. Conclusions

In this study, we observed the volatile production patterns of *A. hydrophila* using the FF-2A electronic nose. This device detected the volatile changes after 30-min culture in lysogency broth at a concentration of 1.8 × 10^3^ CFU/mL. Our results imply that *A. hydrophila* at a starting concentration of 9.6 × 10^2^ CFU/mL can be detected in <2 h, confirming the detection ability of the device. This is the first report on the detection of *A. hydrophila* on the basis of its volatile production patterns with an electronic nose. In future, combination of selection medium and volatile pattern database from microorganisms will increase the detection specificities.
